# Discovering the Giant Nest Architecture of Grass-Cutting Ants, *Atta capiguara* (Hymenoptera, Formicidae)

**DOI:** 10.3390/insects8020039

**Published:** 2017-03-28

**Authors:** Luiz Carlos Forti, Ana Paula Protti de Andrade, Roberto da Silva Camargo, Nadia Caldato, Aldenise Alves Moreira

**Affiliations:** 1 Laboratório de Insetos Sociais-Praga, Departamento de Produção Vegetal, Faculdade de Ciências Agronômicas/UNESP, Caixa Postal 237, 18603-970 Botucatu-SP, Brazil; luizforti@fca.unesp.br (L.C.F.); anapaulaprotti@gmail.com (A.P.P.d.A.); nacbiol@gmail.com (N.C.); 2 Laboratório de Entomologia, Departamento de Fitotecnia e Zootecnia, Universidade Estadual do Sudoeste da Bahia (UESB), , Caixa Postal 95, 45083-900, Vitória da Conquista-BA, Brazil; aldenise.moreira@gmail.com

**Keywords:** grass-cutting ants, social insects, nest

## Abstract

*Atta capiguara* is a grass-cutting ant species frequently found in Cerrado biome. However, little is known about the giant nest architecture of this ant. In this study, we investigated the architecture of three *A. capiguara* nests from a fragment of Cerrado in Botucatu, São Paulo, Brazil. Casts were made of the nests by filling them with cement to permit better visualization of internal structures such as chambers and tunnels. After excavation, the depth and dimensions (length, width, and height) of the chambers were measured. The results showed the shape of *Atta capiguara* nests consisting of mounds of loose soil with unique features resembling a conic section. The fungus chambers were found distant from the mound of loose soil and were spaced apart and distributed laterally at the soil profile. The waste chambers were located beneath the largest mound of loose soil. Both the fungus and waste chambers were separated and distant. Our study contributes to a better understanding of the so far unknown nest architecture of the grass-cutting ant *A. capiguara*.

## 1. Introduction

Nests of species of the genus *Atta* are structurally the most complex nests in the Attini tribe. For example, nests of the species *Atta laevigata* contain more than 7000 chambers and measure up to 8 m in depth [1]. Internally, the nests differ in terms of the shape of the waste and fungus chambers and their location in relation to the external area [2]. In *A. sexdens* nests, the fungus and waste chambers have a semi-ellipsoidal shape and the waste chambers have arm-like prolongations. Both types of chambers are found below a mound of loose soil [2]. In *A. laevigata* and *A. bisphaerica*, the fungus chambers are spherical [1,3]. However, the authors found no waste chambers in any of the excavated nests of these two species.

An *Atta* nest is consequently much larger than the documented nests of other ant genera, such as those of *Pogonomyrmex badius* which contain 150 chambers, *Solenopsis invicta* which builds nests with numerous chambers connected by tunnels in a highly complex and elaborated system, *Pheidole morrissi*, *Prenolepsis impairs*, and *Conomyrma* [4,5]. Even though *Acromyrmex* is the other Attini genus, they do not build nests in comparable size to the genus *Atta* [6,7,8].

Despite this knowledge, the study of the internal architecture of the subterranean nests of ants has received little attention. Although there is a considerable number of studies mentioning the architecture of ant nests, many of them are based on qualitative descriptions accompanied by schematic drawings of the nests [9,10,11,12,13,14,15,16,17,18]. However, quantitative data of ant nests are sparse [1,3,4,16].

The external and internal architecture of the nests of *Atta* and of other ant genera is related to the ecology and behaviour of the species since the nests are considered a superorganism and the individuals behave in such a way that favors the development and specialization of this organism [19]. In this respect, factors such as soil type, ground water table [20,21] and microclimate conditions may significantly affect the depth of ant nests, but the spatial arrangement and design are generally specific for each species [1,3].

In *Atta bisphaerica*, the chambers containing the fungus garden with freshly incorporated plant fragments are found near the soil surface [1,3]. This fact suggests an adaptation of the species to withstand variations in temperature and soil humidity since this species generally builds its nests in open areas with plenty of sunlight where, even in deep soils, chambers are not located at great depths. It therefore seems that nest depth is not limited by the ground water table [1,3]. On the other hand, due to the complexity of the nests, ants choose the best location within the nest in terms of temperature and humidity gradients to rear eggs, larvae and pupae [22,23,24], as well as for the symbiotic fungus and all biota involved [3].

Although several studies have investigated the nest architecture of leaf-cutting ants, addressing external and internal structures, little is known about these structures in grass-cutting ant species, especially *Atta capiguara*. Therefore, the objective of the present study was to provide a detailed description of the nest architecture of the grass-cutting ant, *Atta capiguara*, using cement moulding and excavation.

## 2. Material and Methods

### 2.1. Study Area

The study was conducted at the Santana Farm (20°50′46′′ S; 48°26′2′′ W), located near the Lageado Experimental Farm of UNESP, Botucatu, São Paulo, Brazil in a Cerrado fragment.

### 2.2. Nests Studied

The external area consisting of loose soil was used as a criterion of maturity of the *Atta capiguara* nests. Three nests were used in this study. The size of the nests was confirmed by mapping two or three active entrance holes near the nests. The holes were mapped using plastic straw baits of different colours and cut types (totaling 48 types of baits) according to the method of Fowler et al. ( 1993) [25], modified by Boaretto (2000) [26] for grass-cutting ants.

The straws were cut into 3 to 4 mm pieces and immersed in a solution of concentrated orange juice containing sugar (forming a syrup). The straw fragments were removed with sieves and transferred to trays where a mixture of citrus pulp and sugarcane leaf powder was added. The pieces were carefully moved inside these trays so that the mixture would be well impregnated on the straws and they would be highly attractive to the ants. The straws were dried for 24 h at room temperature. After this period, the baits were stored in plastic bags identified with numbers according to the colour and type of straw to facilitate handling in the field.

In the field, the plastic straw baits were placed in the active holes near the nests. The number corresponding to each type of bait was marked on the stake. After 24 h, the mound of loose soil to which each straw was returned by the ants was identified and the type and colour found, which indicate which entrance holes belong to each nest, were recorded.

After the area covered by the nests was determined, the mound area of the *A. capiguara* nests was measured. These measurements were obtained using the traditional method in which the mound area is calculated by multiplying the greatest length by the greatest width.

### 2.3. Internal Architecture

Three *Atta capiguara* nests (C, C4 and C6) were chosen to visualize the internal architecture and tunnel structure. The nests were moulded using a mixture of 5 kg cement in 10 L of water, which was poured into all open holes above the soil mound of the nest (small mound: live zone; large mound: dead zone) and into the holes spread on the soil surface (foraging holes) as described by Moreira et al. 2003 and 2004) [1,3]. Approximately 3550 kg cement was used for the complete nest cast. The water–cement mixture was poured into the holes using aluminum funnels. Excavation was started 7 days after application of the cement.

Prior to excavation, the external area of the nest was determined by the traditional method, which consists of measuring the greatest width and greatest length of the area comprising all mounds of loose soil (largest mound and rosettes). Two nylon strings were then stretched over the nest area, forming two orthogonal axes (x; y), whose center corresponded to the center of the nest area. These axes were used to identify the chambers and tunnels.

The areas of the nests filled with cement were excavated using small manual tools to avoid their destruction. A 0.70-m wide and 1-m deep ditch was opened around the nest area and excavation was performed from the outside to the inside. The ditch was deepened until complete appearance of the chambers, which were numbered for subsequent measurement. After excavation, the chambers and tunnels were measured and photographed. The following parameters were obtained: dimension (width, height, and length), depth from the soil surface, and position on the orthogonal axes (x; y). The mean, standard deviation and range of the measurements were calculated for statistical analysis.

The volumes of the fungus and waste chambers were calculated and compared to geometric figures. Although they were not perfect figures, the models that best fitted the shape of each type of chamber were used. The formulas proposed by Forti (1985) [27] were applied, i.e., the volume formula for cylinders to the fungus chambers and the volume formula for cones to the waste chambers: 

Volume of fungus chamber = πr^2^ (hc + r0.67), where r = radius of the base of the chamber, and hc = height of the cylinder. The radius was subtracted from the maximum height of the chamber (h) to obtain hc, i.e., h − r = hc. The volume of the waste chambers was estimated by V = πr^2^h/3, where r = radius and h = height.

Since all fungus garden chambers were moulded with cement, the correction factor (1.37) obtained by Forti (1985) [27] was used for comparison between the true volume of the chambers and the volume estimated with the geometric formula to correct the calculated volume. Forti (1985) did not estimate the true volume of the waste chambers because of their large volume. Thus, only the volume estimated with the geometric formula was obtained for this type of chamber.

## 3. Results and Discussion

The *Atta capiguara* nests had the typical shape consisting of several mounds of loose soil with unique features resembling a conic section [28,29]. The external area of the nests measured 221.4 m^2^ (C), 102.4 m^2^ (C4) and 64.8 m^2^ (C6).

**Figure 1:**
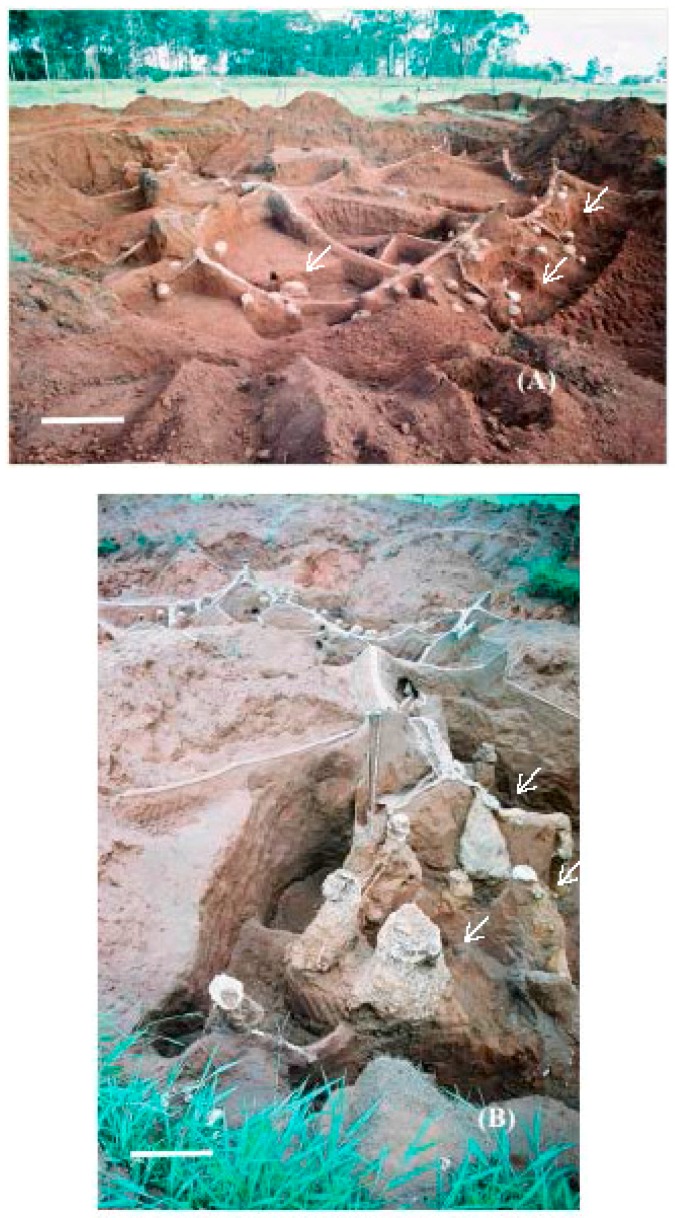
General view of the internal cement-moulded architecture of nest C (*Atta capiguara*): (**A**) area where the fungus chambers are concentrated; see arrows; (**B**) area where the waste chambers are located, see arrows. White bars = 1 m.

**Figure 2:**
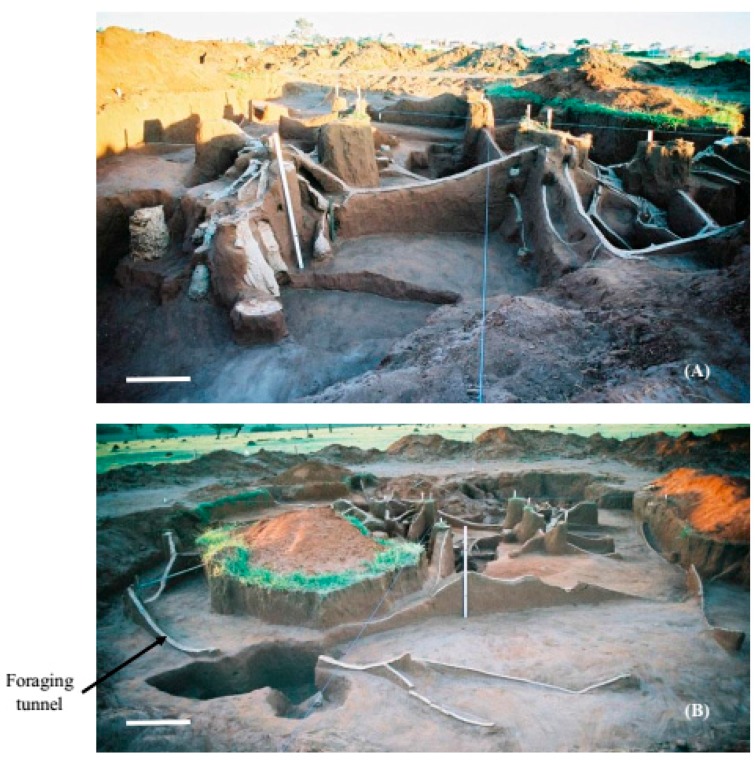
General view of the internal cement-moulded architecture of nest C6 (*Atta capiguara*): (**A**) area where the fungus chambers (right) and waste chambers (left) are located; and (**B**) external ring tunnel connecting one side of the nest to the other. White bars = 1 m.

**Figure 3:**
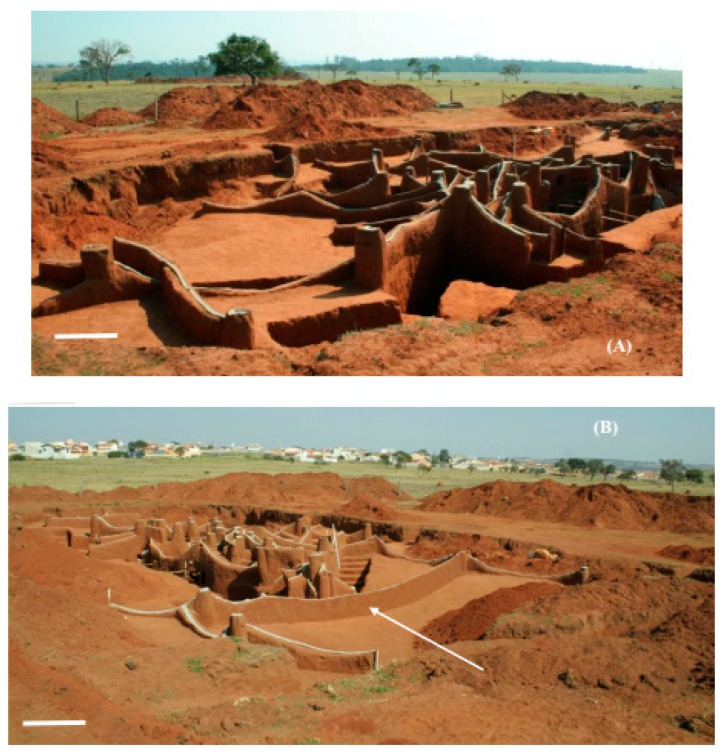
General view of the internal cement-moulded architecture of nest C4 (*Atta capiguara*): (**A**) area where the fungus chambers are concentrated, highlighting the tunnel that connects one end of the nest to the other; and (**B**) area where the waste chambers are located, highlighting the tunnel that laterally connects one side of the nest to the other (white arrow). White bars = 1 m.

The fungus chambers were found distant from the mound of loose soil and were spaced apart and distributed laterally at the soil profile (Figure 1A). The waste chambers were located below the largest loose soil mound (Figure 1, Figure 2 and Figure 3) [27,28,29].

**Figure 4:**
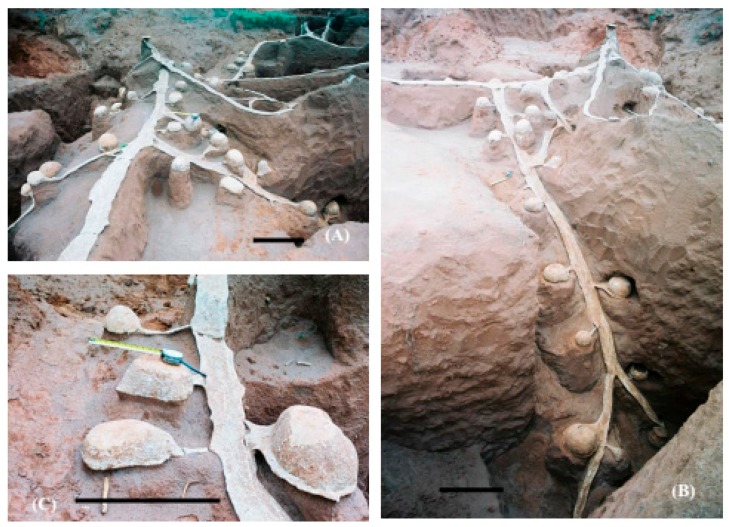
Detail of the tunnels and fungus chambers found in the first *Atta capiguara* nest: (**A**) arrangement of the chambers along one of the branching tunnels lateral to the main tunnel; (**B**) detail of the main tunnel interconnected by chambers and by a branching tunnel; (**C**) detail of fungus chambers connected to their interconnecting tunnels (peduncles) which, in turn, connect to the main tunnel. Botucatu, SP. Black bars = 0.50 m.

**Figure 5:**
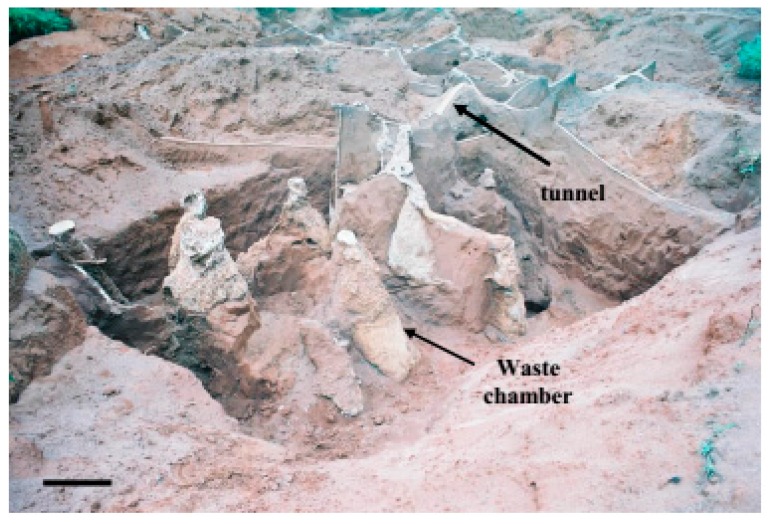
General view of the first excavated *Atta capiguara* nest showing the single interconnection between the areas where the fungus and waste chambers are concentrated. Botucatu, SP. Black bars = 1 m.

In leaf-cutting ant nests, the chambers vary in shape, location and dimension depending on the species and type of chamber (fungus, waste, and soil). The fungus chambers found in the present study had an oval shape with a plane base (Figure 4A) and the waste chambers had a conical shape (Figure 5). Differences according to the type or function of the chambers have also been reported by other authors, such as in *A. capiguara* [27], in *A. sexdens* [2] and in *A. vollenweideri* [20,30,31], with waste chambers differing in shape from the other chambers. An interconnecting tunnel (peduncle) located in the median portion or close to the base was observed in most fungus chambers (Figure 4C). Besides, the remarkable difference between the fungus chambers and the waste chamber is the smoothness of the chamber walls. The waste chambers appear to be rougher and more irregular (Figure 5). The arrangement of the fungus chambers of *A. capiguara* differed from that of other *Atta* species. There was no network of tunnels or a concentrated chamber area resembling a “bunch of grapes” as observed in other species for which studies on nest architecture by cement moulding are available, such as *A. sexdens* [32], *A. laevigata* [1], and *A. bisphaerica* [3].

In the first nest excavated (C), the fungus chambers were connected through a main tunnel (Figure 4A,B). This tunnel was continuous and opened into small mounds of loose soil, i.e., above ground that were part of the external architecture of the nest. Five tunnels branched laterally from this main tunnel (Figure 4A) where additional fungus chambers were found. The chambers that connected to the main tunnel were not very deep and were found at an average depth of 1.2 m (SD ± 0.42) from the soil surface. The most superficial chamber in this area was located at a depth of 0.54 m.

The chambers connected to the tunnels that branched along the main tunnel were found at an average depth of 2.1 m (SD ± 1.0) from the soil surface (Figure 4B). The deepest chamber was located at a depth of 4.2 m in one of the branched tunnels. Forti (1985) [27] also observed chambers at this depth, but it remains unknown whether these chambers were located in branched tunnels or in the main tunnel because the excavated nests were not moulded.

In nests C6 and C4, the main tunnel was not as prominent as in the first nest excavated and the chambers were generally connected to branched tunnels which, in turn, connected to the main tunnel (Figure 2 and Figure 3). In nests C6 and C4, the fungus chambers were found at a lower average depth than in the first nest excavated, with a depth of 1.98 m (SD ± 0.44) from the soil surface in nest C4 and of 1.64 m (SD ± 0.70) in nest C6. However, fungus chambers were found at a depth of 5.85 m from the soil surface in nest C4, while in nest C6 the deepest chamber was located at a depth of 3.35 m. In the first excavated nest, the tunnels connecting to the chambers, including the main tunnel and the branching tunnels, were wide with a flattened shape and contained a rectangular section (Figure 4A,B).

The main tunnel was much wider than the branching tunnels (Table 1). The connection between these wide tunnels (main and branching tunnels) and the fungus chambers was not direct. The chambers connected to these tunnels through small tunnels, also called peduncles (Figure 4C). These peduncles were generally elliptical in cross-section (Figure 4C) and their width and length were reduced, but their height was similar to that of the other tunnels (Table 1). The main and branching tunnels of nests C6 and C4 exhibited the same pattern as those of the first nest.

In the first excavated nest, the areas where the fungus and waste chambers were concentrated was interconnected through a single tunnel (Figure 5). However, three tunnels interconnecting these two nest areas were found in nest C6 (Figure 3), while only one tunnel interconnected these areas in nest C4. In this nest, it was possible to observe a tunnel leading to a foraging hole, which extended throughout the nest until the area where the waste chambers were concentrated (Figure 3A). In the three nests excavated, the waste chambers were also interconnected through a main tunnel and some chambers were connected to each other. The dimensions of the chambers are shown in Table 2, Table 3 and Table 4.

In the first moulded nest, the waste chambers were found at a depth ranging from 0.43 to 1.6 m. In nest C6, the waste chambers were located at similar depths (0.7 to 1.75 m), while in C4 these chambers were found in deeper layers of 2 to 2.6 m from the soil surface. The waste chambers were quite large (Table 2, Table 3 and Table 4; Figure 5), with dimensions—3 m in height and 1.5 m in width—in any of the nests [28,29].

The volume of one of the chambers was much higher (535.5 L) than the total volume estimated for 11 waste chambers (166.5L) [27]. However, the maximum volume observed in nests C6 and C4 was 75.4 and 77.9 L, respectively (Table 3 and Table 4).

The tunnels of the three excavated nests were flat and elliptical in cross-section, but not as wide as the tunnels connecting to the waste chambers. The first moulded nest and nest C6 exhibited the same architectural pattern. In contrast, some differences in the arrangement of the tunnels were found in nest C4. The fungus chambers of this nest were located in deeper soil layers and were concentrated. Another difference was the large number of foraging tunnels, a fact that makes the outer ring not very visible. Nevertheless, it could be observed that the linear tunnel in this nest starts at one end, passes through the fungus chamber area and extends to the area called the dead zone where the waste chambers are concentrated. This tunnel measured 23.3 m in length from one end of the nest to the other (Figure 3A).

However, it is possible that nest C4 was on the decline. There was no open entrance hole on the small soil mounds or on the largest mound (waste), and no fresh soil had been deposited recently on these mounds. We observed no signs of ant activity at this nest site during the daytime.

However, as seen in nest C6, when the cement was poured into the three holes studied, workers reopened several foraging holes around the nest and on the rosettes and largest soil mound.

Casting permitted the demonstration of a peculiar architecture of the nest built by this ant species that was very different from the nests of previously studied species [20,21]. Since this species builds nests in regions characterized by marked solar insolation, i.e., grassland areas, it was reasonable to assume that the fungus chambers are built in deeper soil layers. This hypothesis was not completely confirmed. Although some chambers were found at a depth of up to 5.85 m from the soil surface, in the branching tunnels, a large number of chambers was observed at a depth of slightly more than 1 m that were connected to the main tunnel.

Another interesting finding was that only few chambers were found in the area where the foraging tunnels were concentrated (Figure 1, Figure 2 and Figure 3). Did the workers dig these tunnels that subsequently opened at ground level only to collect plant material for the colony? Associating the fact of having an area in the nest where only foraging tunnels exist, one may state that, in addition to serving for the transport of leaves to the nest, these tunnels are fundamental for the air circulation dynamics inside the nest [33,34,35].

**Figure 6:**
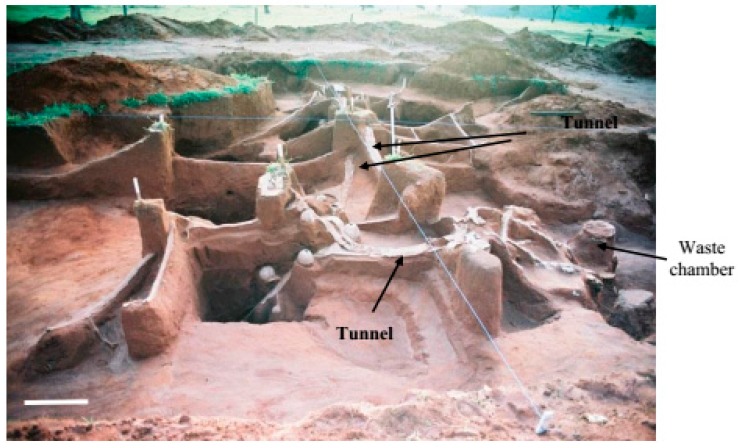
General view of nest C6. Note the three tunnels that connect the area of the fungus chambers with that of the waste chambers. Botucatu, SP. White bar = 1 m.

Casting of the interior of the nests provided another important finding. The connection between the area of the fungus chambers and the area of the waste chamber was made by a single tunnel in the first excavated nest and in nest C4 (Figure 3A and Figure 5), while in nest C6, this connection was made by three large tunnels (Figure 6).

**Figure 7:**
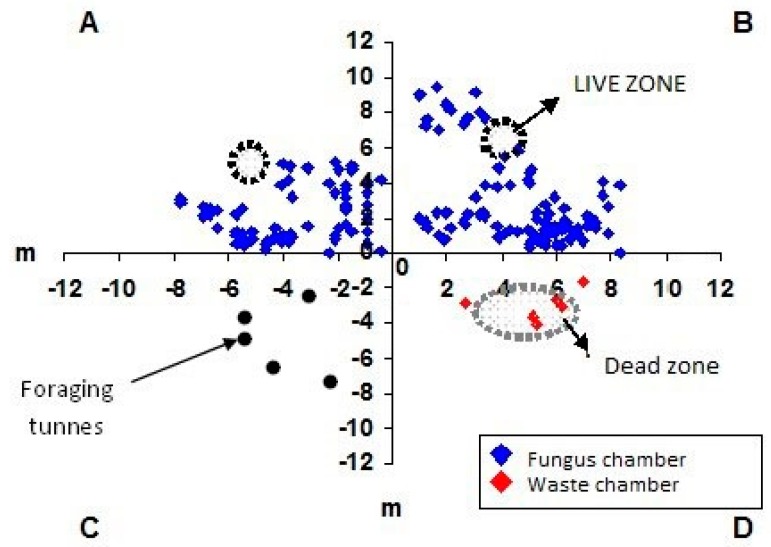
Schematic map of the limits of loose soil (dotted circles) and of the area where the chambers are concentrated in the first nest of *Atta capiguara.*

**Figure 8:**
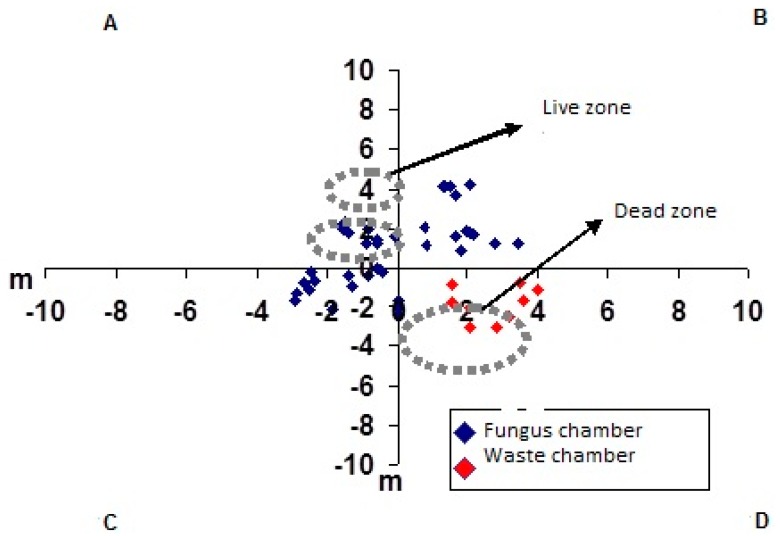
Schematic map of the limits of loose soil (dotted circles) and of the area where the chambers are concentrated in the second nest of *Atta capiguara.*

**Figure 9:**
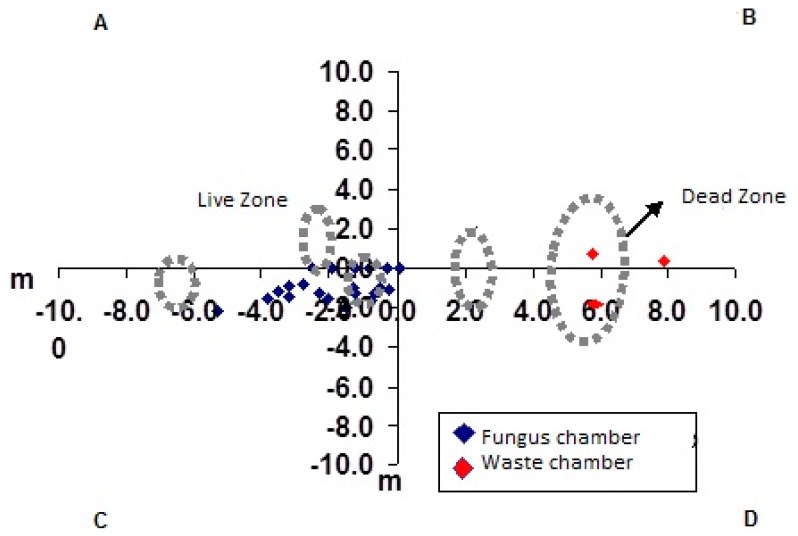
Schematic map of the limits of loose soil (dotted circles) and of the area where the chambers are concentrated in the third nest of *Atta capiguara.*

The little contact between the two nest areas (live and dead zone) (Figure 7, Figure 8 and Figure 9) is interesting and advantageous for the species because it reduces the contact between the area of waste deposition, which contains microorganisms that are harmful to the fungus, and the chamber area where workers cultivate the symbiotic fungus [36,37]. However, in nest C6, fungus chambers were found very close to the waste area, with one of the tunnels connecting one area to the other (Figure 6), this might be due to colony age.

The region where the nests were excavated has been used in previous studies investigating other *Atta* species and it is therefore possible to estimate the age of these nests. Nest C6 was the youngest nest, with a maximum of 5 years. The first excavated nest (C) was 8 to 10 years old and nest C4 was the oldest, with more than 10 years. Since nest C6 was the youngest, it still had several connections with the area where the waste chambers were concentrated. As the nest becomes older, the distance between the two nest areas increases. Nevertheless, the level of specialization of this species seems to be high when compared to other *Atta* species that do not separate the area of waste deposition from the fungus garden.

## 4. Conclusions

Atta capiguara nests can be identified by conical mounds of loose soil visible on the soil surface. The fungus chambers were found, through excavation, to be distant (6.0 m +2.0) from those conical mounds of loose soil and were spaced (8.67 m +1.15) apart at different depths (3.20 m +1.91) in the soil profile.

The waste chambers were located beneath the largest mound of loose soil. Both the fungus and waste chambers were separated and distant. Our study contributes to a better understanding of the so far unknown nest architecture of the grass-cutting ant *A. capiguara*.

## Figures and Tables

**Table 1:** Dimensions of the internal tunnels of the first moulded nest of Atta capiguara. Botucatu, SP.

Type	Width (cm)				Height (cm)				Length (m)			
	Mean	SD *	Max.	Min.	Mean	SD	Max.	Min.	Mean	SD	Max.	Min.
Foraging Tunnels	8	3.5	12	0.6	1.3	2.3	2	1	5.3	1.5	7.0	4.4
Tunnels connecting to the chambers	11	4.1	21	1.3	1.6	0.4	3.6	0.5	4.4	2.3	7.4	0.72

* SD: standard deviation.

**Table 2:** Dimensions of the chambers found in the first moulded nest of *Atta capiguara.* Botucatu, SP.

Chambers	Height (m)				Width (m)				Volume (L)			
	Mean	SD **	Max.	Min.	Mean	SD	Max.	Min.	Mean	SD	Max.	Min.
Fungus (n * = 72)	0.14	0.003	0.23	0.08	0.21	0.06	0.37	0.0006	5.7	4.0	17.5	0.9
Waste (n = 10)	1.4	0.57	2.7	0.6	0.32	0.11	0.53	0.16	172.9	146.6	535.5	20.7

* n: number of chambers found; ** SD: standard deviation.

**Table 3:** Dimensions of the chambers found in nest C6 of *Atta capiguara.* Botucatu, SP.

Chambers	Height (m)				Width (m)				Volume (L)			
	Mean	SD **	Max.	Min.	Mean	SD	Max.	Min.	Mean	SD	Max.	Min.
Fungus (n * = 40)	0.17	0.5	0.38	0.8	0.21	0.5	0.32	0.08	7.5	5.5	22.3	0.8
Waste (n = 8)	0.8	0.3	1.1	0.7	0.14	0.04	0.4	0.16	32.07	39.8	77.9	5.24

* n: number of chambers found; ** SD: standard deviation.

**Table 4:** Dimensions of the chambers found in nest C4 of *Atta capiguara.* Botucatu, SP.

Chambers	Height (m)				Width (m)				Volume (L)			
	Mean	SD **	Max.	Min.	Mean	SD	Max.	Min.	Mean	SD	Max.	Min.
Fungus (n * = 28)	0.13	0.4	0.2	0.6	0.18	0.7	0.4	0.1	4.5	4.9	23.1	0.5
Waste (n = 8)	0.68	0.21	0.8	0.4	0.47	0.08	0.60	0.15	43.55	33.05	75.4	9.42

* n: number of chambers found; ** SD: standard deviation.
